# New Insights into Phasmatodea Chromosomes

**DOI:** 10.3390/genes8110327

**Published:** 2017-11-17

**Authors:** Thomas Liehr, Olesya Buleu, Tatyana Karamysheva, Alexander Bugrov, Nikolai Rubtsov

**Affiliations:** 1 Institute of Human Genetics, Jena University Hospital, Am Klinikum 1, D-07747 Jena, Germany; 2 Novosibirsk State University, 630090 Novosibirsk, Russia; buleu.olesya@mail.ru (O.B.); bugrov04@yahoo.co.uk (A.B.); rubt@bionet.nsc.ru (N.R.); 3 Institute of Cytology and Genetics, Siberian Branch of Russian Academy of Sciences, 630090 Novosibirsk, Russia; kary@bionet.nsc.ru; 4 Institute of Systematics and Ecology of Animals, Siberian Branch of Russian Academy of Sciences, 630090 Novosibirsk, Russia

**Keywords:** stick insects, Phasmatodea, C-banding, fluorescence in situ hybridization, ribosomal deoxyribonucleic acid, telomeric repeats, interstitial telomeric sequences

## Abstract

Currently, approximately 3000 species of stick insects are known; however, chromosome numbers, which range between 21 and 88, are known for only a few of these insects. Also, centromere banding staining (C-banding) patterns were described for fewer than 10 species, and fluorescence in situ hybridization (FISH) was applied exclusively in two *Leptynia* species. Interestingly, 10–25% of stick insects (Phasmatodea) are obligatory or facultative parthenogenetic. As clonal and/or bisexual reproduction can affect chromosomal evolution, stick insect karyotypes need to be studied more intensely. Chromosome preparation from embryos of five Phasmatodea species (*Medauroidea extradentata*, *Sungaya inexpectata*, *Sipyloidea sipylus*, *Phaenopharos khaoyaiensis*, and *Peruphasma schultei*) from four families were studied here by C-banding and FISH applying ribosomal deoxyribonucleic acid (rDNA) and telomeric repeat probes. For three species, data on chromosome numbers and structure were obtained here for the first time, i.e., *S. inexpectata*, *P. khaoyaiensis*, and *P. schultei*. Large C-positive regions enriched with rDNA were identified in all five studied, distantly related species. Some of these C-positive blocks were enriched for telomeric repeats, as well. Chromosomal evolution of stick insects is characterized by variations in chromosome numbers as well as transposition and amplification of repetitive DNA sequences. Here, the first steps were made towards identification of individual chromosomes in Phasmatodea.

## 1. Introduction

Today approximately 3000 species of stick insects, also known as walking sticks (Phasmatodea), are known. An interesting feature of Phasmatodea is that 10–25% of the known species are either obligatory or facultative parthenogenetic [1]. Notably, walking sticks may present with low effective population sizes (e.g., *Peruphasma schultei*) [2]. Additionally, even in tropical habitats, walking stick eggs can require several months to develop, while individual animals live on average 1–2 years [3]. Theoretical work suggests that longer lifespans like this might be associated with higher cell turnover rates [4]. In practice, these factors may lead either to more chromosome abnormalities per generation or a higher likelihood of fixing chromosome abnormalities even if they are slightly deleterious.

Genome sizes of Phasmatodea are close to that of orthopteran grasshoppers [5], which arose ~40.5 million years ago [6]. However, Phasmatodea are much older, and many subgroups evolved in this order in the last ~200 million years [7]. Chromosome numbers in this group are highly variable, and polyploidy is well documented in parthenogenetic taxa. Interestingly, in this group of insects, large genome sizes up to 8 picogram per haploid genome have been observed. The diploid chromosome number in stick insects ranges from 21 (male) or 22 (female), found in several species, up to 88 in *Carausius furcillatus* [2,7]. Also, polyploid species with structurally diploid complements have been reported [8,9]. Thus, many unique features of genome architecture and inheritance patterns can be observed in Phasmatodea. However, cytogenetic studies on them are principally hampered by high chromosome numbers (polyploidy) or poor chromosome morphology, or both.

Of special interest for basic research is how chromosomal evolution and regulation work during parthenogenetic and clonal reproduction are typical for many species of stick insects. In some species, parthenogenetic populations may be observed in parallel to sexual populations [10]; how this is regulated and if and how they interact is still not known. Additionally, it needs to be considered that parthenogenetic reproduction may lead to diversity and selection of the most adapted clones. This has been shown by the breeding of stick insects over generations: some cultures reproduced successfully and stayed phenotypically unchanged, while others underwent unexpected changes. For example, changes in normally red wing color to white or pink in *Peruphasma schultei* in captivity have been reported recently [11]. In a parthenogenetic lineage, new genetic and karyotypic variants normally do not derive from the recombination of the existing genetic and karyotypic elements within the population. Considering the possibility of different egg maturation mechanisms, as observed in *Clonopsis gallica*, restricted recombination may be possible also here [12]. Still, in most cases parthenogenetic reproduction can be suggested to diminish possible genetic diversity, reducing genotypic and phenotypic variability for natural selection. It should be expected that this kind of evolutionary scenario leads to genomes adapted to long parthenogenetic reproduction, and possibly an accumulation of harmful mutations. In this context, structural chromosome re-patterning, such as fissions, translocations, and polyploidy have been observed [13,14,15]. Thus, one major adaptation of parthenogenetic strains is probably whole or segmental polyploidy or amplification of some genomic elements, or both. In alternate or intermixing parthenogenetic and sexual reproducing populations, sexual reproduction can address problems in correct chromosome pairing and recombination during meiosis. This could require special genome and karyotype organization, preventing multivalent formation and ensuring genetic balance. 

Even though only comparably scanty data are available on karyotypes and chromosome structure of stick insects, it is known that there are different sex chromosome systems in Phasmatodea. Most stick insects have, besides numerous autosomes, only one gonosome—i.e., one X chromosome. This X0-system is most likely the ancestral sex chromosome system (68 taxa, present in 36 of 46 studied genera). The second largest group of Phasmatodea species exhibits an XY sex chromosome system (13 species, eight genera) [15]. Additionally, *Didymuria violescens* has males with both X0 and XY complements. Out of 95 studied bisexual species, nine were found with XX/XY, while in the others an XX/X0 sex chromosome system was observed [14]. 

Another peculiar karyotypic feature of stick insects relates to the presence/absence and different numbers and sizes of cytological visible satellites, varying among co-generic and even within the same species [16,17,18,19,20]. For two species from the genus *Leptynia*, *Leptynia montana* (2n = 38/37; XX/X0) [21], *L. attenuate* (2n = 36/36; XX/XY) (subspecies *L. attenuata attenuate*, *L. attenuata iberica*, *L. attenuata algarvica*) regions enriched for 45S ribosomal deoxyribonucleic acid (rDNA) and telomeric repeats (TTAGG)_n_ were reported [22].

Despite an increasing interest in Phasmatodea genetics, cytogenetic studies are not very frequent, compared to other model insects such as *Drosphila*. This may be due to problems in obtaining chromosomes, as laboratory cultures of stick insects often consist only of parthenogenetic females. Accordingly, karyotyping is not an easy task, as the most convenient and useful material for cytogenetic analysis in insects is male testes. Ovarioles can also successfully be used for chromosome preparation, but they contain only a few dividing cells—in our experience, only one of four laboratories used to prepare animal and/or specifically insect chromosomes, was able to obtain stick insect chromosomes for the present study. 

Chromosomes of Phasmatodea were up to now mainly studied by routine staining approaches (i.e., Giemsa). Even centromere banding staining (C-banding) of stick insect chromosomes was described only for a few species [23,24]. Molecular cytogenetics, i.e., fluorescence in situ hybridization (FISH) in stick insects, was applied in a single study [22]. Thus, here we intended to provide karyotypic characterization of the following five species from four families applying C-banding, and to characterize the distribution of 18S rDNA and telomeric repeats by FISH:

Phasmatidae:

*Medauroidea extradentata* (Vietnam) is a species first reported in 1907 [4]; it can be bisexual in captivity, but also parthenogenetic. Cytogenetics was previously done resulting in a karyotype of 2n = 38 [25]; later others [10] reported a karyotype 37,X0 for male.

Heteropterygidae:

A parthenogenetic population of *Sungaya inexpectata* (Philippines) was discovered and described for Luzon Island in 1996; in 2008, on the same island a bisexual population of this species was found [26]. No karyotypic data have been published yet.

Diapheromeridae:

*Sipyloidea sipylus* (Southeast Asia; also, due to humans, Madagascar) was first described in 1859 [27], and established lab strains are mainly parthenogenetic; in the wild, bisexual populations can also be found. The first description of its karyotype was published in 1967 [28]. In females the chromosome number was determined initially to be 80 ± 2 and it was suggested that *S. sipylus* is tetraploid [28]. Later, the chromosome number in *S. sipylus* was listed as 4n = 80 [29].

*Phaenopharos khaoyaiensis* (Thailand) was first described by Zompro around 1999 [30]; in culture, this species again is only parthenogenetic. In the wild, sexual reproduction is possible. No karyotype has been published yet.

Pseudophasmatidae:

*Peruphasma schultei* (Peru) was discovered in 2002 and first described in 2005 [3]. It is a bisexual species, found only on 5 hectares of land as an endemic species. No cytogenetic data are available.

## 2. Material and Methods

### 2.1. Stick Insects Studied

Five species of stick insects were maintained and bred as laboratory cultures: *Medauroidea extradentata* (Phasmatidae) [4], *Sungaya inexpectata* (Heteropterygidae) [26], *Sipyloidea sipylus* (Diapheromeridae) [27], *Phaenopharos khaoyaiensis* (Diapheromeridae) [30], and *Peruphasma schultei* (Pseudophasmatidae) [10]. Only *P. schultei* presented with a bisexual population in our setting. The other four species included exclusively parthenogenetic females. *M. extradentata*, *S. inexpectata*, *S. sipylus*, and *P. khaoyaiensis* were fed with rasp- and blackberry leaves, *P. schultei* with privet (Ligustrum) leaves. All of them were kept on their fodder plants up to the cytogenetic analyses. 

### 2.2. Chromosome Preparation

Thirty- to 40-day-old embryos from stick insect eggs were used for the preparation of metaphase chromosomes—initial preliminary work (our own unpublished data) indicated that eggs of this age were best suited for chromosome preparations. The embryos were dissected out of the eggs and placed in a 0.9% sodium citrate solution at room temperature. For 20–30 min they were fixed in a cold methanol:glacial acetic acid mixture (3:1). Fixed embryos were macerated in a drop of 60% acetic acid on slides and air-dried [31,32]. The per-species available metaphases for the present study are listed in Table 1 (~2 metaphases per embryo; in *M. extradentata* the amount was doubled).

Meiotic chromosome preparation was performed from testes of *P. schultei* according to standard protocols. Males were injected with 0.1 mL of 0.1% colchicine in double-distilled water for 1.5–2.0 h prior to fixation of testes in ethanol:glacial acetic acid (3:1) for 15 min, and then kept in 70% ethanol. Air-dried chromosome preparations were obtained by squashing testis follicles in 45% acetic acid and then freezing them on dry ice.

### 2.3. C-Banding

C-positive regions were visualized by C-banding as described by Sumner [33]. 

### 2.4. DNA Probe Preparation

The probe for (TTAGG)_n_ telomeric DNA was generated by polymerase chain reaction (PCR) with the oligonucleotides (TTAGG)_5_ and (CCTAA)_5_ as primers as described [34]. Labeling was achieved by additional PCR cycles replacing dTTP (=desoxythymidintriphosphat) by Tetramethyl-Rhodamine-5-dUTP (Tamra-5-dUTP). For detection of 18S rDNA a fragment of human 18S rDNA cloned in pHr13 165 [35] was applied. The rDNA-probe was labeled with Alexa 488-5-dUTP by nick-translation according standard protocols [36].

### 2.5. Fluorescence In Situ Hybridization

Two-color FISH was carried out as described [36], with small modifications. Briefly, chromosome preparations were denatured in 70% formamide/2x saline-sodium citrate (SSC) solution for 5 min at 75 °C, and dehydrated in standard series of precooled ethanol at −20 °C; the DNA probes in hybridization mixture were denatured separately for 5 min at 95 °C; then hybridization was carried out overnight in a humid chamber at 37 °C. After hybridization, slides were washed under low stringency conditions (3 times for 5 min each, in 50% formamide/2xSSC at 45 °C, 2xSSC at 45 °C and 0.2xSSC at 45 °C). Counterstaining was done by Vectashield Antifade Mounting Medium (Vector Laboratories, Burlingame, CA, USA) containing 4′,6-diamidino-2-phenylindole (DAPI) (Vector Laboratories) applied directly under a coverslip that was then sealed with rubber cement.

### 2.6. Microscopy

Microscopy and imaging were done at the Centre for Microscopy of Biological Objects (Institute of Cytology and Genetics, Novosibirsk, Russia) with an AxioImager.M1 (Zeiss, Jena, Germany) fluorescence microscope equipped with #49, #46HE, #43HE filter sets (Zeiss), ProgRes MF (JenaOptik, Jena, Germany) CCD camera. Software package ISIS5 version (MetaSystems, Altlussheim, Germany) was used for image acquisition and analysis. 

## 3. Results

In four species, *M. extradentata*, *S. inexpectata*, *S. sipylus*, and *P. khaoyaiensis* chromosome analyses were performed only on embryos obtained from parthenogenetic females. Chromosome numbers were determined, patterns of C-banding described, and FISH with a labeled fragment of 18S rDNA and telomeric repeats was carried out. In *P. schultei*, additionally to chromosomes from embryos, meiotic chromosomes of two males were analyzed. 

### 3.1. Medauroidea extradentata

**Figure 1:**
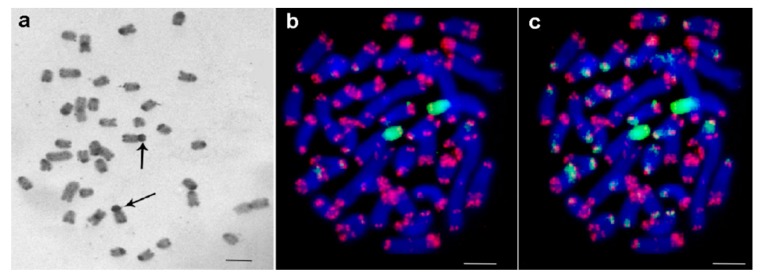
Metaphase plates of female embryo of *Medauroidea extradentata*. (**a**) C-banding: large submetacentrics have in their short arms proximal C-positive regions, indicated by arrows is thepair with the largest C-positive regions; (**b**) Fluorescence in situ hybridization (FISH) with 18S ribosomal deoxyribonucleic acid (rDNA) (green) and telomeric repeats (red) showed that the in (**a**) highlighted chromosome pair has rDNA accumulated in C-positive region. Chromosomes were counterstained by 4′,6-diamidino-2-phenylindole (DAPI) (blue). (**c**) The same metaphase as in (**b**), but with longer exposure time for green channel, is shown. Other chromosomal regions also gave weak signals using 18S rDNA specific probe. Scale bar indicates 5 μm.

The chromosome number published earlier for this species was 2n = 38 for female [25]. We could confirm that the karyotype contained two pairs of metacentrics, one pair of submetacentric and 16 acrocentric chromosomes. C-banding of *M. extradentata* is shown in Figure 1a; in detail: in the pericentric regions of all chromosomes apart from the largest acrocentric chromosome, large- or medium-sized C-positive regions were observed. Intercalary C-positive regions were also discovered in two pairs of acrocentrics. In the majority of acrocentrics and in all metacentrics small dot-like C-positive regions were identified in terminal regions of long (=q)-arms. The most impressive C-banding pattern was discovered in a pair of large submetacentrics. Their short arms consisted of large proximal C-positive regions, flanked by a small C-negative region and the small dot-like C-positive region on the tip of the chromosome arm; an identical dot-like C-positive region was also present on the end of its long arm. 

**Figure 2:**
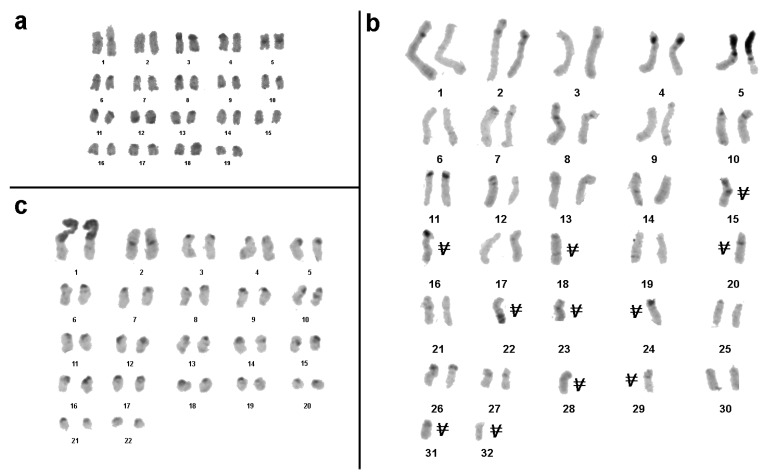
Karyograms from metaphase plates of female embryos of three of the studied species; gonosomes could not be identified. (**a**) Female *M. extradentata* have 19 chromosome pairs, i.e., most likely a karyotype 36,XX. (**b**) For female *Sungaya inexpectata* no complete metaphases could be obtained; here we show one incomplete metaphase with 53 chromosomes and 32 different chromosome pairs identified—lacking chromosomes are indicated by a V. Still, it is obvious that this species is diploid. (**c**) Female *Sipyloidea sipylus* have 22 chromosome pairs, i.e., most likely a karyotype 42,XX.

Interestingly, FISH with the labeled fragment of 18S rDNA stained the whole short arm of the latter mentioned submetacentric chromosome (Figure 1b). FISH signals of the labeled 18S rDNA on these chromosomes were very strong and made it difficult to observe signals in other chromosomal regions (Figure 1b). Nevertheless, an additional small FISH signal could be observed near the termini of some other chromosomes. For detailed analysis, the level of the weak signal was artificially increased (Figure 1c). In this way, small clusters of 18S rDNA were detected in numerous sites. They varied in size and were colocalized or localized near to the clusters of telomeric repeats. Additionally, interstitial clusters of telomeric repeats (Interstitial Telomeric Sequences—ITSs) were present in many chromosomes. Usually they were located not far from clusters of telomeric repeats in the termini of long arms. In some chromosomes, they were close to the clusters of telomeric repeats in the termini of long arms and were almost fused with them (Figure 1b,c). In the future, the pattern of the here applied probes together with other repetitive DNA probes might be used to clearly identify individual chromosome pairs. An attempt to do this based on C-banded chromosomes is shown in Figure 2a.

### 3.2. Sungaya inexpectata

**Figure 3:**
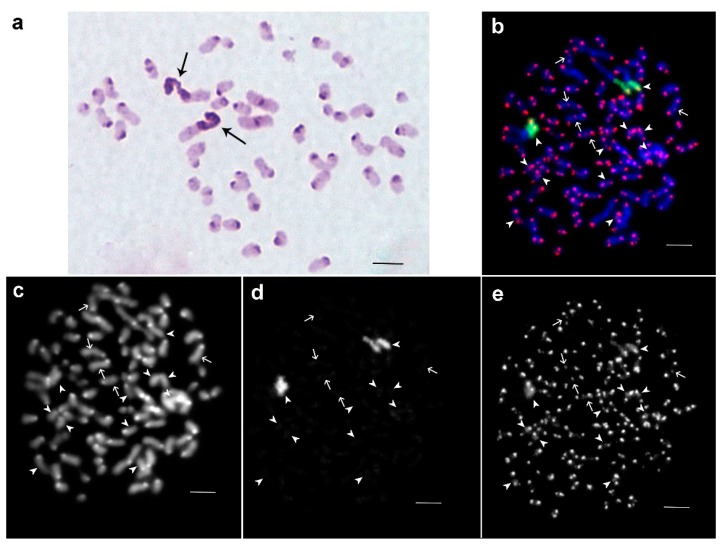
Metaphase plates of female embryo of *S. inexpectata*. (**a**) C-banding: large metacentrics contain C-positive arms (arrows); (**b**) FISH as in Figure 1b; shown are late metaphase chromosomes (chromatids of many chromosomes were separated); (**c**) DAPI staining; (**d**) FISH with 18S rDNA only; (**e**) FISH with labeled telomeric repeats only. The arrowheads show the regions the regions containing the dispersed telomeric repeats. The arrows show interstitial telomeric sites. Scale bar indicates 5 μm.

*S. inexpectata* had 44 chromosomes per metaphase plate including two pairs of large metacentric, and 20 pairs of acrocentric chromosomes of different sizes. Pericentric C-positive regions were present in all chromosomes. One arm of the largest metacentric chromosome pair was C-positive (Figure 3a). Two-color FISH showed that the C-positive region on this chromosome pair was enriched for 18S rDNA; telomeric repeats were also present in this region. The latter were dispersed along the heterochromatic region, but due to their small numbers they produced a FISH signal of lower intensity (Figure 3b–e). On other chromosomes, clusters of telomeric repeats were located at the chromosomal tips. Additionally, ITSs were observed in C-negative regions of some chromosomes (Figure 3c,e), and besides the classical clusters of telomeric repeats dispersed telomeric repeats were also observed in pericentric C-positive regions of some chromosomes (Figure 3c,e) of *S. inexpectata*. For the karyotype see Figure 2a.

### 3.3. Sipyloidea sipylus

**Figure 4:**
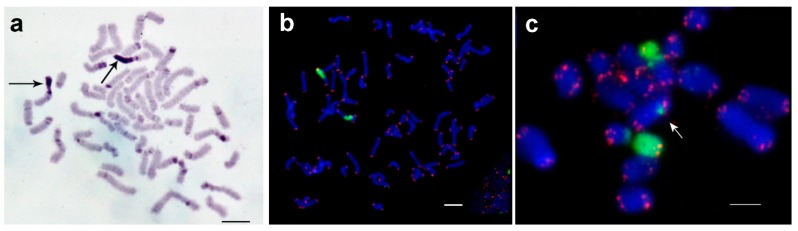
Metaphase plates of a female embryo of *S. sipylus*. (**a**) C-banding: large metacentrics contain large C-positive regions in one arm (arrows); (**b**) FISH as in Figure 1b; (**c**) FISH as in Figure 1b on a partial metaphase plate. Scale bar indicates 5 μm.

Karyotype of *S. sipylus* was earlier described [28] and chromosome number determined as 4n = 80 published by More [29]. Despite the selection of metaphase plates for chromosome analysis according to features suggesting their unbrokenness, the chromosome number in metaphase plates varied in this study from 53 to 71. The chromosome plates of analyzed embryos included two pairs of large metacentrics, remaining chromosomes were submetacentics, varying in size from medium to small. Besides pericentric C-positive regions, C-bands were present in telomeric position of some chromosomes. In different chromosomes, they were in short or long arms. In a few chromosomes, interstitial C-positive regions were also identified. One pair of the large metacentrics was characterized with a large C-positive region. It was separated by a pericentric C-band with a small C-negative region similar in size to the short arm of medium acrocentrics (Figure 4a). According to the data obtained here this species is not tetraploid (Figure 2b).

In the majority of studied embryos, two-color FISH revealed that exclusively the large C-positive regions of the aforementioned chromosome pair were enriched for 18S rDNA (Figure 4b,c). Besides these chromosomes, only one embryo had an additional chromosome containing a small cluster of 18S rDNA (Figure 4c). 

Further clusters of telomeric repeats in chromosomes of *S. sipylus* were discovered only at chromosomal ends and no ITS were identified (Figure 4b). 

### 3.4. Phaenopharos khaoyaiensis

**Figure 5:**
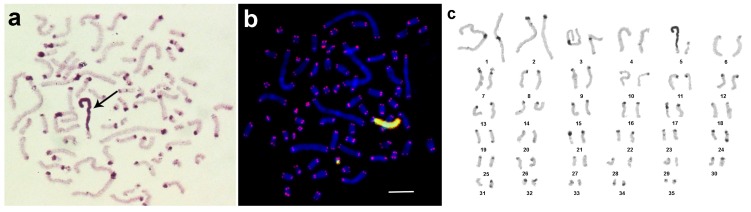
Metaphase plates of a female embryo of *Phaenopharos khaoyaiensis*. (**a**) C-banding: a large C-positive region is visible (arrow); (**b**) the C-positive region is highlighted by FISH (18S rDNA, green) and homologue chromosome is also stained. Scale bar indicates 5 μm; (**c**) Female *P. khaoyaiensis* have 70 chromosome pairs, i.e., most likely a karyotype 34,XX.

For *P. khaoyaiensis* the chromosome number was 70 for the majority of analyzed cells(Figure 2c), thus, a female karyotype of 34,XX is suggested. In a minority of cells, only 68 chromosomes were detectable, which was most likely an artifact. The analyzed chromosome plates of *P. khaoyaiensis* included one large, one medium and one small pair of metacentric chromosomes, while the remaining chromosomes were acrocentrics or acrocentrics containing a small short arm. C-positive regions were localized in the pericentric regions of almost all chromosomes. Moreover, in pericentric regions, C-positive stretches were also detected in terminal and intercalary positions of some chromosomes. Among all analyzed embryos, one large chromosome including a large C-positive arm and a small C-negative arm was observed. According to C-banding pattern, no chromosome homologous to it was found (Figure 5a). Subsequent two-color FISH revealed that the large C-positive long arm was enriched for telomeric repeats and repeats homologous to the fragment of 18S rDNA. Furthermore, an erstwhile unidentifiable homologous chromosome was found (Figure 5b). In chromosome spreads of *P. khaoyaiensis* there were two chromosomes containing cluster of 18S rDNA. They showed the same C-negative arms similar in size, but one of them contained a small short arm, while the other contained a large C-positive arm, both of them enriched for 18S rDNA and telomeric repeats (Figure 5b). In Figure 5c a karyotype is presented; gonosomes were not identified.

### 3.5. Peruphasma schultei 

**Figure 6:**
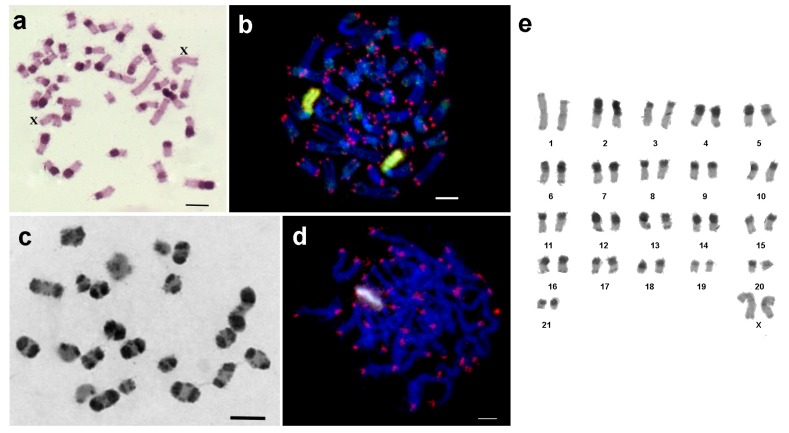
Mitotic and meiotic chromosomes of *Peruphasma schultei*; (**a**) C-banding of embryonal metaphase chromosomes. The X chromosomes are labeled with ‘X’. (**b**) FISH as in Figure 1b. (**c**) C-banding of adult male derived meiotic chromosomes. (**d**) FISH on meiotic chromosomes (pachytene), using probes as given in Figure 1b. Scale bar indicates 5 μm; (**e**) Female *P. schultei* have 22 chromosome pairs and a karyotype 21,XX.

In *P. schultei* 20 metaphase plates contained 44 chromosomes (44,XX) and two of them contained 43 chromosomes (43,X0). The female karyotype of *P. schultei* consisted of two pairs of large metacentrics, one pair of medium metacentrics and one pair of large submetacentrics. Remaining chromosomes were medium and small submetacentrics and acrocentrics. In contrast to the female, the male karyotype contained only three large metacentrics, suggesting that the X chromosome in this species is the large metacentric. Small pericentric and telomeric C-positive regions were found in all large metacentrics (Figure 6a). The medium sized metacentric contained large pericentric C-positive region in the short arm and small C-positive region in the terminal position of the long arm. The large submetacentric had a C-positive short arm, and in the long arm, there were a large pericentric C-band and a small C-band in the terminal position. Other chromosomes showed large pericentric C-positive regions (Figure 6a). 

Two-color FISH showed that the short arm of the large submetacentric chromosome was enriched for 18S rDNA and telomeric repeats, while ITSs were not found along the whole karyotype. 18S rDNA probe painted slightly some of the large pericentric C-bands in other chromosomes, as well (Figure 6b).

Meiotic chromosomes were prepared from the testes of two males. They formed 21 bivalents and one univalent (X) (Figure 6c,d). C-banding technique revealed in autosome bivalents C-bands as described above. In the X chromosome, small C-bands were visible at telomeric position in both arms while in the pericentric region no C-positive region was detectable (Figure 6c). FISH with the labeled fragment of 18S rDNA and telomeric repeats intensively painted one large extended region on pachytene chromosomes. This corresponded to the short arm of a large submetacentric chromosome. Other regions were painted with labeled 18S rDNA only slightly. On pachytene chromosomes, no ITSs were found (Figure 6d). In Figure 5e, a karyotype is presented; gonosomes were identified here. 

## 4. Discussion

To the best of our knowledge, this is only the second study on stick insects applying FISH techniques [22] and here we added one more C-banding study. Five species were studied, only two of which had been karyotyped previously. The presented results provide new insights on Phasmatodea, as outlined below.

### 4.1. Does Parthenogenetic Reproduction Lead to New Lineages Characterized by Chromosomal Evolution?

A long series of parthenogenetic generations, as present in at least four of the studied species, could lead to an accumulation of genetic abnormalities, including chromosome mutations. Considering this, the variations in chromosome numbers observed for *S. sipylus* and *P. khaoyaiensis* may be due to laboratory linages with such chromosomic features or to technical problems. Indeed, embryos were used to obtain chromosomes, thus, selected eggs may have genomic imbalances, which would have been lethal in the egg or shortly after hatching. Thus, the questions of chromosome number variation and the probable structural rearrangement in polyploid species of stick insects in culture remain open for the moment. 

Recently, elevated levels of aneuploidy were described in laboratory lines of free-living flat worms *Macrostomum lignano* [37,38]. During its evolutionary history this species has undergone whole genome duplication followed by chromosomal rearrangements [39]. In this species, chromosome abnormalities might have disturbed the delicate gene balance, but instead did not lead to any abnormal development or pathological phenotype. Thus, it is at least possible that, in parthenogenetic females of polyploid species, phenotypically normal linages characterized by chromosome abnormalities can arise. These lineages can be an evolutionary dead end or can generate new diversity for further natural selection and evolution. Application of molecular in situ approaches for reliable karyotyping of individual samples of Phasmatodea is required to answer the question of comparative molecular cytogenetics of different strains of polyploid species in stick insects.

### 4.2. Does the Diversity of C-Banding Patterns in Different Taxa Indicate Intense Amplification and/or Transposition of Mobile Elements as Well as Different Types of Repeats during Chromosomal Evolution?

The diversity of the C-band patterns in the studied species can be discussed concerning: (a) the location and size of C-positive regions, (b) the DNA content of C-positive regions, and (c) the large C-positive regions identified in all studied species, including bisexual *Leptynia* (Pantel) [21,22].

For (a): All the studied species diverged a long time ago and went through numerous changes during their evolutionary history, involving repeat amplifications leading to C-positive regions and elimination of amplified repeats as well. In all the species we studied, C-positive regions were present in pericentric, telomeric, and interstitial positions of different chromosomes; the only exception was *S. inexpectata*, where no C-bands could be visualized in the termini of long chromosome arms. Also, in all the species studied here, at least one chromosome pair had a large C-banding positive region. Thus, overall, there are common features in C-band patterns in the stick insects we studied.

For (b): Yet the DNA content of most C-positive regions in Phasmatodea is not clear. Thus, in the future, it would be very useful to estimate the DNA homology of telomeric C-bands in different Phasmatodea species. This task could be solved by chromosome microdissection of telomeric C-bands of one species followed by generation and labeling of a DNA probe by degenerate oligonucleotide-primed polymerase chain reaction (DOP-PCR) or whole-genome amplification (WGA) techniques. Other approaches using low copy repeats as probes [40,41] or high-throughput approaches are also helpful in this context [42]. The telomeric C-bands in *P. khaoyaiensis* chromosomes are the most promising regions for the suggested microdissection approach. They are large and present in many chromosomes and there are chromosomes with telomeric C-bands in the short or long arms, and in both. It will also be possible to compare the DNA content in such C-bands and in pericentric C-bands, and among the different chromosomes.

For the correct functioning of a centromere, it must be flanked by clusters of repeats of 500 kb or more [43,44]. We suppose that a failure in detection of C-bands in the pericentric region is due to their small sizes or may be due to technical issues with C-banding. In *P. khaoyaiensis* chromosomes, there are C-bands in distal regions separated by small C-negative regions (Figure 5a). The approach that has been suggested before of producing microdissection-derived probes would allow for estimating the homology of their DNA. In the case of revealed homology, it can be an argument in favor that more proximal C-bands derive from terminal ones by transposition of repeats or by a small inversion. Interestingly, the chromosomes of *P. khaoyaiensis* are characterized by many C-bands located close to each other. 

Also, in the future, the suggested stick insect-derived microdissection libraries could be sequenced to clarify the genetic content of C-bands, as was previously shown [45]. Later, DNA probes homologous to the identified repeat sequences could help us to understand the distribution of these repeats in C-bands [45]. Yet, only some of the large C-positive regions of stick insects could be shown to contain rDNA and, in some species, to be enriched with telomeric repeats [22].

For (c): The identification of the mentioned large C-positive region leads to the following questions: Why did all species of studied stick insects show the large rDNA regions? Why were there only two of them per diploid genome? 

The presence of the rDNA-positive C-positive regions underlines two surprising points: the formation and maintenance of this region itself, and its presence in metaphases of five distantly related taxa, i.e., the five species studied here, and two species in *Leptynia* [22]. Previously, large telomeric regions enriched for rDNA and telomeric repeats were discovered in voles, i.e., *Sorex granarius* [46]. These regions contain more than 300 kb of discontinued telomeric repeats, localized on short arms of acrocentric chromosomes. In some telomeric regions, the sets of telomeric repeats alternate with repeats of rDNA and other DNA sequences. In the termini of other chromosomes, the sets of telomeric repeats were located a little further away from the clusters of rDNA [47]. The possible mechanisms of formation of these terminal regions include unequal crossing-over and amplification of these repetitive DNA arrays [48]. These very large telomeres were detected only in one vole species, *Sorex granarius*. In a closely related species, *Sorex araneus*, the telomeres were of conventional size [49]. The large C-positive regions in stick insects showed some analogy and at the same time differences from the large *Sorex granarius* telomeres. Like *Sorex granarius* telomeres, they contain regions enriched for rDNA or both rDNA and telomeric repeats. Furthermore, in *S. inexpectata* this region is enriched by telomeric repeats to a smaller extent. It is unknown if the arrays of (TTAGG)_n_ repeats in this species are shorter or their number was decreased. These regions were found both in parthenogenetic females (Figure 1, Figure 2, Figure 3, Figure 4 and Figure 5) and in bisexual species (Figure 6) [22]. For stick insects, we can suggest mechanisms involved in the formation of these large C-positive regions, like unequal crossing over, or DNA amplification in somatic cells, as is involved in the formation of a homogeneously staining region [50]. 

The most surprising feature of large C-positive regions containing rDNA in stick insects is the sheer size and their localization on a single chromosome pair. According to the present study, there are taxa including species with a few nucleolus organizing regions (NORs) per karyotype (most of the species studied here), while other taxa have species characterized by numerous NORs of distinct size (*M. extradentata*; Figure 1c). However, even in the first type of taxa, NOR locations are on different chromosomes also in related species, suggesting the transposition of rDNA followed by its amplification at the new spot and elimination of the old NORs. Also, in the three species studied here, and also in *Leptynia* [22], the large C-positive blocks are also enriched in telomeric repeats. Interestingly, in *S. sipylus* a large C-positive block with rDNA and telomeric repeats is present only on one chromosome; in addition, there is a smaller chromosome, with a smaller correspondingly stained region (Figure 4a–c). Either these two chromosomes are different autosomes, or this pattern is a hint of an unusual sex-chromosome system, one being the X and the other the Y chromosome.

The large enriched regions of rDNA or rDNA and telomeric repeats suggest that there are special mechanisms for the restriction of rDNA and telomeric repeat distribution along chromosomes in stick insects. However, chromosomes of *M. extradentata* are different. The chromosomes of this species are characterized by two large rDNA regions, but other chromosomes also contain many small clusters of rDNA and ITSs. These additional small clusters of rDNA are located mostly near the centromeric regions, while additional clusters of telomeric repeats appear close to the end of long arms. Some clusters of telomeric repeats are located very close to each other. Also, in the FISH results they look almost like fused dots. Other clusters are separated by regions of assorted sizes, suggesting inversion-transferred telomeric repeats in more proximal positions. There are also ITSs inside the chromosomes. Two different mechanisms of ITS formation are known. One of them suggests chromosome rearrangements involving telomeres. The second suggests repair of DNA damage by telomerase [51]. Irregular distribution of ITSs along chromosome arms with a concentration near the chromosomal ends make us hypothesize that most of them are a result of chromosome rearrangements that took place during the chromosomal evolutionary history of *M. extradentata*.

In our samples, the most intense DNA amplification, leading to large C-positive regions besides the large regions enriched by rDNA or rDNA and telomeric repeats, appears to have taken place during the chromosomal evolution of *P. schultei.* Large C-bands are present in the pericentric regions of all chromosomes apart from a pair of large metacentric autosomes and the X chromosome. Some of them form whole chromosome arms in biarmed chromosomes; in other biarmed chromosomes, they are even present in both arms, whereas in acrocentrics, C-bands were only observed in pericentric regions. In *P. schultei*, the chromosome morphology after C-banding makes the identification of centromeres difficult for many chromosomes. However, in DNA amplification forming C-bands, a few copies of rDNA genes or fragments homologous to them were involved and visualized by FISH (Figure 6b). 

## 5. Conclusions

In stick insects, despite chromosome evolution providing a vast variety of chromosome numbers, probably involving polyploidy and whole genome duplication, all the studied species contain in their karyotypes large regions enriched by rDNA or rDNA and telomeric repeats. Besides rDNA and telomeric repeats, other DNA sequences were also amplified, providing different patterns of C-bands in the chromosomes of stick insects.

This study is a first step towards the identification of individual chromosomes in Phasmatodea. This will enable further, more detailed studies on chromosomal evolution in this abnormal group of insects. 

## Figures and Tables

**Table 1:** Number of metaphase plates per analyzed species.

Species	Embryos Prepared	Metaphases Obtained	Metaphases per Embryo
*Medauroidea extradentata*	28	112	4.0
*Sungaya inexpectata*	68	107	1.7
*Sipyloidea sipylus*	15	28	1.9
*Phaenopharos khaoyaiensis*	31	59	1.9
*Peruphasma schultei*	10	22	2.2
